# Intelligent Traffic Model for Unmanned Ground Vehicles Based on DSDV-AODV Protocol

**DOI:** 10.3390/s23146426

**Published:** 2023-07-15

**Authors:** Ali M. Ali, Md Asri Ngadi, Israa Ibraheem Al_Barazanchi, Poh Soon JosephNg

**Affiliations:** 1 Department of Computer Science, Faculty of Computing, University Technology Malaysia, Johor Bahru 81310, Malaysia; 2 Computer Engineering Techniques Department, Baghdad College of Economic Sciences University, Baghdad 10, Iraq; 3 College of Engineering, University of Warith Al-Anbiyaa, Karbala 65001, Iraq; 4 Faculty of Data Science & Information Technology, INTI International University, Persiaran Perdana BBN, Nilai 71800, Negeri Sembilan, Malaysia

**Keywords:** DSDV-AODV, VANET, V2X, QoS, UGV

## Abstract

Traffic systems have been built as a result of recent technological advancements. In application, dependable communication technology is essential to link any system needs. VANET technology is used to communicate data about intelligent traffic lights, which are focused on infrastructure during traffic accidents and mechanisms to reduce traffic congestion. To ensure reliable data transfer in VANET, appropriate routing protocols must be used. This research aims to improve data transmission in VANETs implemented in intelligent traffic lights. This study investigates the capability of combining the DSDV routing protocol with the routing protocol AODV to boost AODV on an OMNET++ simulator utilizing the 802.11p wireless standard. According to the simulation results obtained by analyzing the delay parameters, network QoS, and throughput on each protocol, the DSDV-AODV routing protocol performs better in three scenarios compared to QoS, delay, and throughput parameters in every scenario that uses network topology adapted to the conditions on the road intersections. The topology with 50 fixed + 50 mobile nodes yields the best results, with 0.00062 s delay parameters, a network QoS of 640 bits/s, and a throughput of 629.437 bits/s. Aside from the poor results on the network QoS parameters, the addition of mobile nodes to the topology influences both the results of delay and throughput metrics.

## 1. Introduction

Recent technological advancements have enabled the development of traffic systems, which require dependable communication technology in order to function. VANET technology is used to communicate data about intelligent traffic lights, which are focused on improving infrastructure during accidents and reducing traffic congestion. In order to ensure reliable data transmission, suitable routing protocols must be used in VANET. The globe is facing the challenges of increased traffic, pollution, and traffic accidents. One of the major issues produced by a significant number of transport vehicles is the emergence of traffic congestion at city junctions. Current technical advancements have led to the creation of an intelligent traffic system called intelligent traffic light. This technology improves the efficiency of traffic management, monitoring, and control in terms of cost, performance, and maintenance. To communicate with UGV networks in the use of intelligent traffic lights, dependable communication network technology is required. Aside from the advancement of wireless networks, several technologies have evolved to improve the efficiency and effectiveness of communication. The Vehicular Ad Hoc Network (VANET) and its interaction with routing protocols are two developing technologies. Previously, the authors of [1,2,3] performed a study on the full potential of the convergence of VANET and WSN on the Internet of Things for urban data collecting. Additionally, some experiments [4,5] using VANET to handle file transfers use data communication on intelligent traffic lights, emphasizing the interface infrastructure. Some reviewers provided an overview of various computing paradigms related to vehicular networks. It outlined the key features of each computing paradigm and highlighted the open research challenges in vehicular networks. Choosing the correct routing protocol in VANET is important in establishing reliable data transfers, especially given the necessity for a network design that adapts to demands and situations. According to the research of [6,7], who investigated Dedicated On-Demand Routing Protocols (AODV), Dynamic Source Routing (DSR), and Destination Sequenced Distance Vector Routing Protocol (DSDV) on VANET, DSDV and AODV routing protocols are the recommended routing protocols, and they have the best performance when compared to other routing protocols. Based on this background, research is required to compare AODV and DSDV-AODV routing protocols to examine a reliable intelligent traffic light data transmission system on a chassis built using VANET network technology. However, because the ad hoc attribute is difficult to understand and difficult in forecasting an application’s performance when it is implemented for a network, one effective technique to analyze this system is to simulate it in real time. Much research on network simulation tools [8,9,10] has shown that the Optimized Mobile Network Engineering Tool (OMNET++) is the best solution for executing VANET simulations. Researchers can simply extract metric parameters on the network because the OMNET++ emulator supports most VANET protocols. As a result, this study’s contribution is to simulate and implement data transmission on intelligent traffic lights utilizing the DSDV-AODV routing protocol on the VANET. Because of its simulation employing wireless network technology, the OMNET++ simulator employs the 802.11b wireless standard, which has long-term advantages and is now more frequently used than the 802.11a standard [11,12,13]. Some researchers have evaluated the performance of AODV, DSR, and DVR routing protocols, where the simulation results showed that AODV was more efficient, but DSR achieved the best results in terms of energy consumption [14]. In addition, some researchers have studied a new way to integrate proactive and reactive routing in ad hoc networks [15]. In addition, the network topology was altered in this study based on the road conditions at the junctions to investigate the delay, network QoS, and throughput measurement parameters of AODV and the improved DSDV-AODV routing protocol. It is intended that the findings will be utilized as a basis for future studies on the use of VANET to intelligent traffic signals. The aim of this research is to enhance data transmission in VANET used in intelligent traffic signals. Specifically, the study will focus on the development of a new communication protocol that can reduce delays and increase the efficiency of data delivery. The contribution of this study suggests that combining DSDV and AODV routing protocols can improve the performance of AODV in terms of packet delivery ratio, average end-to-end delay, and average control overhead. The study also shows that the proposed combination of DSDV and AODV routing protocols yields better results in terms of throughput and the packet delivery ratio compared to AODV or DSDV alone. Furthermore, the proposed approach can be used to improve the performance of various routing protocols in Vehicular Ad Hoc Networks (VANETs). The remainder of the paper is structured as follows: The approach is presented in Section 2. Section 3 contains the results and discussion. Finally, in Section 4, the conclusions are presented. Table 1 represents the abbreviations used in the proposed study.

## 2. Method

This section aims to present a comprehensive overview of how to utilize an OMNET++ simulator to develop, test, and analyze a Vehicular Ad Hoc Network (VANET) simulation. First, the necessary theoretical foundations are laid out in order to form the basis of the proposed research framework. The simulation model is then designed and implemented with OMNET++. Finally, the simulation is tested and analyzed with a selection of empirical tools in order to validate its performance.

### 2.1. Proposed UGVs

Unmanned Ground Vehicles (UGVs) are becoming increasingly popular in the field of Vehicular Ad Hoc Networks (VANETs). UGVs are able to navigate autonomously through their environment, making them ideal for providing efficient, low-cost solutions to urban traffic problems. UGVs can be used to monitor and control traffic flow, detect objects in the environment, and provide real-time navigation guidance to drivers. Additionally, UGVs can be used to aid in the implementation of smart traffic lights. Smart traffic lights can be used to reduce traffic congestion, prioritize emergency vehicles, and improve overall safety. By using UGVs to monitor the traffic flow, smart traffic lights can adjust the signals accordingly. This can help reduce the amount of time a driver spends at a red light, minimize travel time, and improve overall efficiency. UGVs are also being used to detect and identify obstacles in the environment, such as pedestrians, cyclists, and other vehicles. This can help improve the safety of drivers by providing early warnings of potential hazards. UGVs can also be used to monitor traffic conditions and provide real-time navigation guidance. This can help reduce the amount of time drivers spend stuck in traffic, as well as reduce the number of emissions.

### 2.2. VANET Proposed Scheme

**Figure 1:**
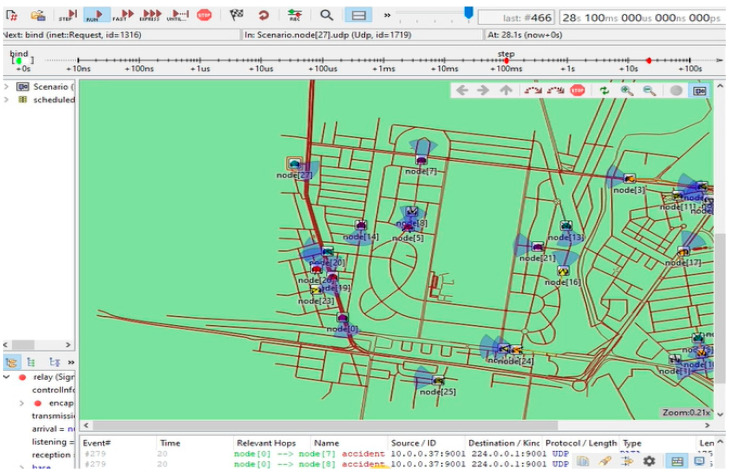
Application of the network simulation model on the OMNET++ emulator.

The simulation was implemented according to the conducted scenarios. Three scenarios were designed to be examined in this simulation, and the results are shown in the Section 3.2. The study consists of several experiments that differ in protocol and topological conditions. The simulation using a Discrete Event Simulator (DES) is a tool called OMNET++ Modeller. This simulation divides into three scenarios based on network sizes utilizing the AODV and DSDV-AODV routing protocols, with V2X network topologies employing fixed nodes and mobile nodes with different numbers. In terms of network design and the simulation model, there is a software environment to design a MANET simulation network according to the list of proposed scenarios, as shown in Figure 1. The figure consists of 20 nodes as an example scenario for six scenarios of iterations: 20 static nodes, 20 static nodes + 20 mobile nodes, 50 static nodes, 50 static nodes + 50 mobile nodes, 500 static nodes, and 500 static nodes + 500 mobile nodes. In this simulated network model, a WLAN-type IEEE 208.11b was used in each scenario configured with the protocol used in the scenario. Table 2 represents the simulation setting parameters and values.

### 2.3. DSDV-AODV

#### 2.3.1. Work Mechanism of the Protocol

**Figure 2:**
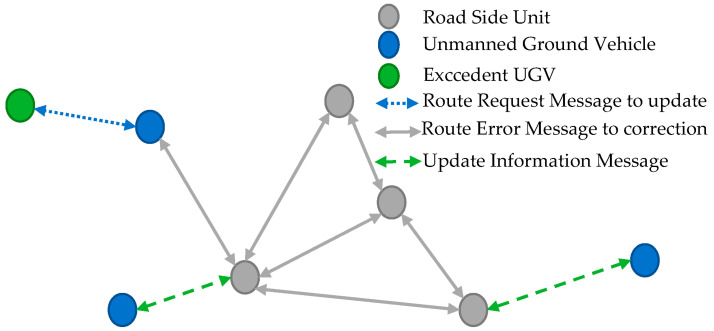
Signal propagation mechanism of the DSDV-AODV protocol.

The DSDV-AODV protocol was designed to send the collision signal as early as feasible in order to rearrange traffic. Many criteria were considered when utilizing VANET routing protocols that used UDP. The suggested protocol is a hybrid of the DSDV (proactive) and AODV (reactive) protocols. The DSDV-AODV protocol is depicted in Figure 2. Each RSU has a routing table that specifies all accessible destinations, the number of hops required to get there, and the node sequence number. To avoid overlap, the sequence number is utilized to distinguish between old and new recordings. The RSU station communicates its routing table to all surrounding RSUs on a regular basis, as well as the schedule in the case of a significant change in the routing table, and therefore depends on the transmission time and intensity of events. If there are two routes in the same sequence, the higher sequence number (the newest) or the best measure (the shortest path) is delivered. Instead of constructing a routing database containing all routes, the AODV routing algorithm improves on the DSDV method by creating a route constraint on demand and the most recent item in the routing table. Instead of the proactive routing table, the interactive routing table is established. A path request packet is broadcast by the collision source or event. The neighbor nodes then broadcast a request to neighbor nodes until the request reaches the target RSU. A route-reply packet is created to respond to the request with only the most recent information. When an event happens, the source sends a Route Request Message (RREQ) to RSU to be included, and the routes are readjusted using a Route Error Message (RRER) until all routes are repaired. A Route Request-Reply (RRER) is a message sent in a Vehicular Ad Hoc Network (VANET) to reply to a Route Request (RREQ) message in order to establish a route between two nodes. The RRER message contains the source address, the destination address, the total hop count, and the hop-by-hop sequence of the route. Algorithm 1 shows the integration of proactive and reactive routing protocols.
**Algorithm 1** Network middleware procedureInput: accident signalOutput: rearrange traffic tableMethod: Hybrid DSDV-AODV protocolProtocol table: all accessible destinations || the number of hops required to get there || the node sequence number.
Receive traffic information period updates   If an accident signal is active then     If the shortest path found in the RSU routing table       ------DSDV (proactive) protocol-------       renew the alternative short path record in the table by replacing the sequence number     Else       -------AODV (reactive) protocol--------       creating a route constraint on demand and the most recent item in the routing table     End If   Else     Notify no change on the cluster   End If

#### 2.3.2. Implementation on V2X

The proposed routing is an ad hoc routing-on-demand AODV routing combined with a DSDV proactive schedule routing. DSDV routing is a type of link-specific routing that is optimized for dedicated mobile networks. This type of proactive protocol follows the principle of the schedule. With the proactive nature of the connection path, it will be provided instantly when two nodes want to connect, due to the routing information that was previously stored in the routing table. This is unlike interactive routing protocols, which will only provide communication methods when needed, as is the case with the AODV protocol. To maintain the routing information in the routing table, every time DSDV needs to update the current route information by dumping broadcast packets on all nodes on the VANET. However, if immersion is performed on all existing nodes, it will cause data overload. To solve this problem, DSDV has a Multipoint Relay (MPR) node that will select only certain nodes to receive broadcast packets, which will be optimized with AODV so that this capability creates a DSDV that has the lowest possible routing overhead compared to other routing protocols. AODV is a routing protocol created for VANET, where this algorithm will create a path between nodes only when desired by the source node. AODV keeps the path for as long as the source node needs it. AODV uses sequence numbers to ensure that the resulting route is loop-free and contains the latest routing information. In AODV, each node is responsible for maintaining the route information that is stored in its routing table. At the time of data transmission, if there is a change in the topology that causes a node to be unreachable using the route information in the routing table, the node will send an RRER path error packet to its neighboring node and the neighboring node will send RRER again and so on until it reaches its destination source node. Each node that receives an RRER will delete the error information in its routing table. Then, the source node will perform the route discovery process again if the route is still required [16,17].

### 2.4. Benchmarks

A routing protocol’s performance is assessed using performance metrics. This statistic utilizes standard units to gauge network performance. Average delay, network QoS, and throughput are some of the metrics utilized in this study to gauge how well the protocol performs in the network.

#### 2.4.1. QoS

Network QoS, also known as network load, is the quantity of routing traffic delivered based on the rate in bits/s at which data packets are sent from the source to the destination, which is calculated as shown in Equation (1). It also controls how many packets are transmitted over the network. In other words, the volume of control messages transmitted to the destination determines the traffic stress. In bits/s, the network load is expressed. Additionally, network load is a situation in which there is a lot of traffic on the network, and it is challenging for the network to handle it all. High network QoS have an impact on packet routing on the VANET by slowing down packet delivery to the target node and increasing packet collisions [18,19].

(1) QoS=P×D+F×T

where the components of the equation are as follows:

*P* = Performance;

*D* = Delay;

*F* = Frequency;

*T* = Throughput.

#### 2.4.2. Delay

When network protocols employ all of their available resources, there is a time delay created by the transmission process from one point to the destination. This performance of the network is known as a delay. An intelligent traffic application is one of those that are sensitive to packet delay. Equation (2) illustrates the many forms of delays, including processing delay (PD), transmission delay (TD), and propagation delay (PD) [20,21].

(2) $$d_{end-end}=N\;(d_{trans}+d_{prop}+d_{proc})$$

where the components of the equation are as follows:

*d_end-end_* = End to end delay;

*d_tran_*_s_ = Transmission delay;

*d_prop_* = Propagating delay;

*d_proc_* = Processing delay.

#### 2.4.3. Throughput

Throughout influences the typical pace at which packets arrive across the transmission line. It assesses protocol performance efficiency and effectiveness and measures network performance from one node to the destination. The ability of the routing algorithm to transport the network is also examined. Throughput is influenced by various variables, such as network architecture changes, erratic communication between nodes, bandwidth, and power restrictions. High throughput is the only way to measure network capability. Additionally, productivity may be quantitatively described as seen in Equation (3) [22,23,24].

(3) $$Throughput=\frac{number\;of\;packet\;sent\times packet\;size\times 8\left(bits\right)}{Total\;simulation\;time}$$

## 3. Result and Discussion

In this section, the proposed simulation of intelligent traffic is evaluated and discussed. The simulation was applied on the 802.11p networks and the DSDV-AODV routing protocol was employed. The results are computed and reviewed to identify the best routing strategy for the measured metric values, based on the scenarios stated in the Section 2.

### 3.1. Results

The OMNET++ simulator produces the simulation results, and all results are presented as charts with data that must be examined and described to provide details of the performance. The amount of time needed for the simulation is called simulation time (ST). This section determines the outcomes of various situations based on the number of nodes in the network layout. The comparison is represented by a table that includes previously examined parameters. The table compares the outcomes of the various employed routing protocols, and the subsections evaluate the results for each scenario.

#### 3.1.1. The First Scenario

This section explains the variations in each parameter’s findings from two scenarios with the same structure but different routing strategies. These scenarios are as follows:

- The scenario in which the network contains 20 fixed nodes. The DSDV-AODV routing protocol is represented by a segmented line. The AODV routing protocol is represented by a continuous line.
- The scenario that has a structure made up of 20 fixed nodes and 20 movable nodes. The DSDV-AODV routing protocol is represented by a segmented line. The AODV routing protocol is represented by a continuous line.

**Figure 3:**
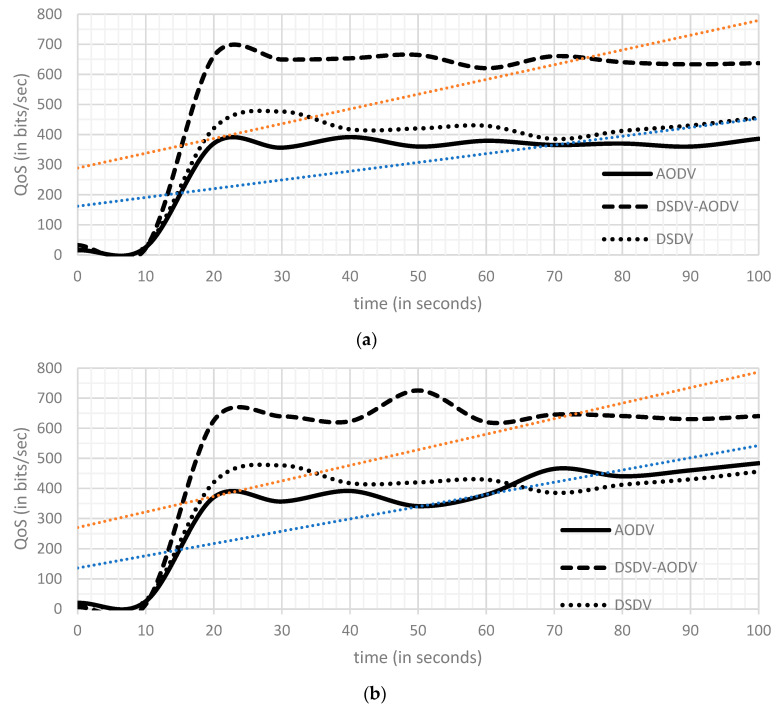
Network QoS graph in scenario 01: (**a**) 20 fixed and (**b**) 20 fixed + 20 mobile.

**Figure 4:**
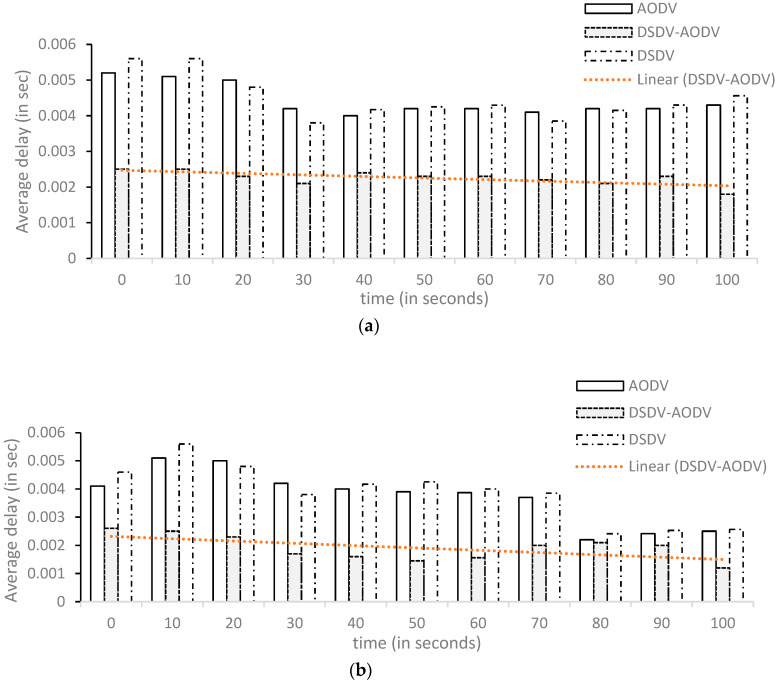
Network delay graph in scenario 01: (**a**) 20 fixed and (**b**) 20 fixed + 20 mobile.

**Figure 5:**
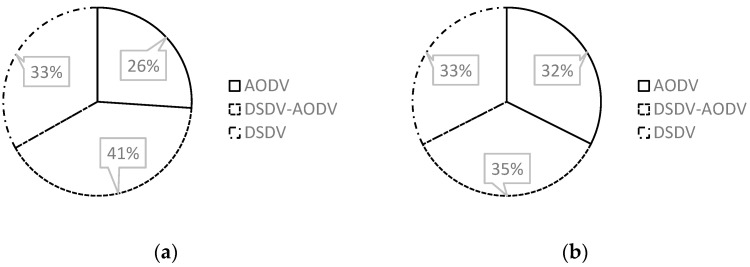
Network throughput graph in scenario 01: (**a**) 20 fixed and (**b**) 20 fixed + 20 mobile.

As seen in Figure 3, Figure 4 and Figure 5, the *X*-axis shows the simulation’s time (in seconds), while the *Y*-axis depicts the levels of delay (in seconds), QoS (in bits/s), and throughput (in bits/s).

##### Comparison of QoS

According to Figure 3, while employing a fixed 20-node topology, the DSDV-AODV routing protocol improves AODV compared to the network QoS settings in the first scenario. Table 3 (a) demonstrates that there was a very significant increase in the outcomes from the start of the scenario to the end. In the second scenario, AODV achieved a lowest network QoS of 386 bits/s and 484 bits/s at 20 fixed and 20 mobile nodes, as shown in Table 3 (b).

##### Comparison of Delay

The performance of DSDV-AODV, which improves AODV by adding DSDV, is shown in Figure 4. The graph demonstrates that routing methods become better when using mobile nodes. AODV has an average delay equal to 0.0043 s with 20 fixed nodes and 0.0025 with the addition of the mobile node, and DSDV routing has an average delay of 0.0046 s using 20 fixed nodes and 0.0026 with the addition of the mobile node topology; the DSDV-AODV routing protocol achieves the best results for the delay coefficient in both cases in the first scenario with an average delay of 0.0018 s in the topology of the static nodes and 0.0012 s with the addition of the mobile node topology. The simulation’s ultimate results are displayed in Table 4 (a, b).

##### Comparison of Throughput

Even if the results are near, Figure 5 depicts the throughput in the first scenario employing fixed nodes on DSDV-AODV, which is best after the experiment. According to Table 5 (a, b), DSDV-AODV yields the greatest results, averaging 629.43 bits per second with fixed nodes and 627.49 bits per second with mobile nodes.

#### 3.1.2. The Second Scenario

This section explains the variations in each parameter’s findings from two scenarios with the same structure but different routing strategies. These scenarios are as follows:

- The scenario in which the network contains 50 fixed nodes. The DSDV-AODV best results are QoS = 126.1, delay = 0.00168, and throughput = 260.53.
- The scenario that has a structure made up of 50 fixed nodes and 50 mobile nodes. The DSDV-AODV results are QoS = 192.47, delay = 0.00062, and throughput = 130.8).

These results are shown in Table 6.

#### 3.1.3. The Third Scenario

This section explains the variations in each parameter’s findings from two scenarios with the same structure but different routing strategies. These scenarios are as follows:

- The scenario in which the network contains 500 fixed nodes. The DSDV-AODV best results are QoS = 69.8, delay = 0.00142, and throughput = 74.1.
- The scenario that has a structure made up of 500 fixed nodes and 500 mobile nodes. The DSDV-AODV best results are QoS = 342.5, delay = 0.00133, and throughput = 372.3.

These results are shown in Table 7.

### 3.2. Result Comparison and Analysis

The behavior of the routing protocol and the average value of each employed metric parameter may be seen from all of the comparison analyses in Section 3.1. The DSDV-AODV routing protocol performs significantly better in nearly every measure across the range. Table 7 demonstrates that the DSDV-AODV delay improves the AODV delay in every circumstance. However, there are some situations where the AODV routing protocol is better than DSDV-AODV for network QoS. In addition to throughput characteristics, DSDV-AODV improves AODV in several scenarios and has a greater throughput. We can determine how each direction will behave in the present environment based on the simulation results that were acquired. The results of each parameter are impacted by the inclusion of mobile nodes, as indicated in Table 7. A mobile node in the scenario reduces the stated delay result in the delay parameter. The smallest average delay, as seen in scenario 02, is 0.00062 s. Additionally, adding mobile nodes to the scenario increases the result value of QoS parameter. Increasing the number of mobile nodes has the effect of improving the results for the throughput metric. Nearly all cases with low delay settings result in high throughput numbers, as shown in Table 8. In addition, the overall simulation results show that the second scenario using 50 fixed nodes + 50 mobile nodes has the best results. Additionally, the results of the total simulation suggest that the second scenario, which uses 50 fixed nodes and 50 movable nodes, produces the best outcomes.

## 4. Conclusions

Based on the implementation, testing, and analysis of data communication simulation and analysis on intelligent traffic lights using AODV and DSDV-AODV protocols applied on 802.11p networks, it can be concluded that the DSDV-AODV routing protocol is superior to AODV based on the results of performance parameters for all three scenarios in the research analysis. In contrast, DSDV-AODV has the smallest average delay (0.00062 s), while AODV and DSDV have the smallest average delays (0.00167 and 0.00157 s) among the values of the second scenario with 50 fixed nodes and 50 mobile nodes. Additionally, the results for the throughput parameter in DSDV-AODV exceed the two other scenarios, with a value of 629.43 bits/s in the first scenario. The lowest AODV routing in the third scenario yields a value for the network QoS parameter of 63.5 bits/s, but the DSDV-AODV routing yields a value of 69.8 bits/s. In comparison to the other two cases, the scenario with 50 fixed nodes and 50 mobile nodes produces the best results for the metric parameters. Although the outcomes for network QoS parameters are unsatisfactory, the inclusion of mobile nodes to the topology improves the node parameter delay and throughput. In the end, it can be said that the contributions of this research suggest a novel model to enhance intelligent traffic lights using the DSDV-AODV protocol for adaptively picking the appropriate routing path for Unmanned Ground Vehicles by using extensive simulations. We verified our analytical QoS, delay, and throughput models, and proved the new enhanced performance of intelligent traffic lights.

The results from this study showed that the proposed combination of DSDV and AODV routing protocols improves the performance of the AODV protocol by reducing the average end-to-end delay and increasing the packet delivery ratio. Additionally, the simulation results revealed that the combination of DSDV and AODV reduces the overall amount of data transmitted in the network. This reduces the overall load on the network and helps to improve the performance of the VANETs. Therefore, the proposed combination of routing protocols has the potential to improve the performance of VANETs implemented in intelligent traffic lights.

## Figures and Tables

**Table 1:** The abbreviations used in the proposed study.

Abbreviation	Description
VANET	Vehicular Ad Hoc Network
DSDV	Destination Sequenced Distance Vector
AODV	Ad Hoc On-demand Distance Vector
QoS	Quality of Service
DSR	Dynamic Source Routing
DVR	Distance Vector Routing
UGV	Unmanned Ground Vehicle
V2X	Vehicle To Everything
WSN	Wireless Sensor Network
DES	Discrete Event Simulator
UDP	User Datagram Protocol
RSU	Road-Side Unit
RREQ	Route Request Message
RRER	Route Error Message
MPR	Multipoint Relay

**Table 2:** Simulation setup parameters.

Parameter	Value
Environment size	1000 × 1000 m^2^
Number of UAVs	200
UAV location	35, 75
Simulation time	3400 s
Transmission range	2450 m
Packet size	50 bytes
Energy for dissipation	100 pJ/bit
Wireless standardization	IEEE802.11P

**Table 3:** Scenario 01 results: QoS parameter (kbps).

(a) 20 Fixed Nodes			(b) 20 Fixed Nodes + 20 Mobile Nodes		
AODV	DSDV	DSDV-AODV	AODV	DSDV	DSDV-AODV
15.638	18.14	32.6	20.8	18.6	6.1
365.35	385.36	660.72	341.35	420.35	725
386.2	455.3	637.5	484.35	456.3	640

**Table 4:** Scenario 01 results: Delay parameter (second).

(a) 20 Fixed Nodes			(b) 20 Fixed Nodes + 20 Mobile Nodes		
AODV	DSDV	DSDV-AODV	AODV	DSDV	DSDV-AODV
0.0052	0.0056	0.0025	0.0041	0.0046	0.0026
0.0042	0.0038	0.0023	0.0039	0.0042	0.0014
0.0043	0.0046	0.0018	0.0025	0.0026	0.0012

**Table 5:** Scenario 01 results: Throughput parameter (kbps).

(a) 20 Fixed Nodes			(b) 20 Fixed Nodes + 20 Mobile Nodes		
AODV	DSDV	DSDV-AODV	AODV	DSDV	DSDV-AODV
44.19	45.19	32.208	95.8	44.19	62
412.96	417.96	741.162	665.2	472.96	719.9
402.63	432.3	629.437	578.7	582.63	627.49

**Table 6:** Scenario 02 results.

(a) 50 Fixed Nodes			(b) 50 Fixed Nodes + 50 Mobile Nodes		
AODV	DSDV	DSDV-AODV	AODV	DSDV	DSDV-AODV
QoS parameter (kbps)					
5.2	6.2	3.67	16.49	17.2	9.42
103.7	107.1	118.9	147.1	152.1	180.5
107.8	112.6	**126.1**	144.29	153.9	**192.47**
Delay parameter (second)					
0.00173	0.00180	0.00169	0.00267	0.0025	0.00026
0.00194	0.00198	0.00190	0.00140	0.00141	0.00078
0.00183	0.00178	**0.00168**	0.00167	0.00157	**0.00062**
Throughput parameter (kbps)					
18.68	20.81	49.4	5.3	8.3	12.7
158.9	161.7	282.6	135.3	138.1	139.6
156.5	168.3	**260.53**	111.39	117.39	**130.8**

**Table 7:** Scenario 03 results.

(a) 500 Fixed Nodes			(b) 500 Fixed Nodes + 500 Mobile Nodes		
AODV	DSDV	DSDV-AODV	AODV	DSDV	DSDV-AODV
QoS parameter (kbps)					
5.16	6.20	1.456	6.97	9.2	23.2
70.8	78.1	84.2	363.97	352.4	385.6
63.5	62.6	**69.8**	328.5	331.9	**342.5**
Delay parameter (second)					
0.00216	0.00218	0.000267	0.00495	0.00425	0.00028
0.00171	0.00169	0.00166	0.00169	0.00164	0.00143
0.00148	0.00147	**0.00142**	0.00192	0.00195	**0.00133**
Throughput parameter (kbps)					
5.1	5.31	5.8	25.1	8.3	40.54
73.9	80.7	88.2	406.2	410.1	412.2
67.2	68.3	**74.1**	367.2	365.39	**372.3**

**Table 8:** Results of all parameters averaged for each scenario.

Parameter	(AODV, DSDV)		DSDV-AODV	
	20 fixed nodes	20 fixed nodes + 20 mobile nodes	20 fixed nodes	20 fixed nodes +20 mobile nodes
QoS (kbps)	(386.2, 455.3)	(484.3, 456.3)	637.5	640
Delay (second)	(0.0043, 0.0046)	(0.0025, 0.0026)	0.0018	0.0012
Throughput (kbps)	(402.63, 432.3)	(578.7, 582.63)	629.437	627.49
	50 fixed nodes	50 fixed nodes +50 mobile nodes	50 fixed nodes	50 fixed nodes + 50 mobile nodes
QoS (kbps)	(107.8, 112.6)	(144.29, 153.9)	126.1	192.47
Delay (second)	(0.00183, 0.00178)	(0.00167, 0.00157)	0.00168	0.00062
Throughput(kbps)	(156.5, 168.3)	(111.39, 117.39)	260.53	130.8
	500 fixed nodes	500 fixed nodes +500 mobile nodes	500 fixed nodes	500 fixed nodes + 500 mobile nodes
QoS (kbps)	(63.5, 64.6)	(328.5, 331.9)	69.8	342.5
Delay (second)	(0.00148, 0.00147)	(0.00192, 0.00195)	0.00142	0.00133
Throughput (kbps)	(67.2, 68.3)	(367.2, 365.39)	74.1	372.3

## Data Availability

Not applicable.
